# Current perspectives on the multiple roles of osteoclasts: Mechanisms of osteoclast–osteoblast communication and potential clinical implications

**DOI:** 10.7554/eLife.95083

**Published:** 2024-04-09

**Authors:** Valentina Daponte, Katrin Henke, Hicham Drissi

**Affiliations:** 1 Department of Orthopaedics, Emory University School of Medicine Atlanta United States; 2 VA Medical Center Atlanta United States; Washington University in St. Louis United States; University of Michigan-Ann Arbor United States

**Keywords:** bone homeostasis, clastokines, communication, coupling, osteoblasts, osteoclasts

## Abstract

Bone remodeling is a complex process involving the coordinated actions of osteoblasts and osteoclasts to maintain bone homeostasis. While the influence of osteoblasts on osteoclast differentiation is well established, the reciprocal regulation of osteoblasts by osteoclasts has long remained enigmatic. In the past few years, a fascinating new role for osteoclasts has been unveiled in promoting bone formation and facilitating osteoblast migration to the remodeling sites through a number of different mechanisms, including the release of factors from the bone matrix following bone resorption and direct cell–cell interactions. Additionally, considerable evidence has shown that osteoclasts can secrete coupling factors known as clastokines, emphasizing the crucial role of these cells in maintaining bone homeostasis. Due to their osteoprotective function, clastokines hold great promise as potential therapeutic targets for bone diseases. However, despite long-standing work to uncover new clastokines and their effect *in vivo*, more substantial efforts are still required to decipher the mechanisms and pathways behind their activity in order to translate them into therapies. This comprehensive review provides insights into our evolving understanding of the osteoclast function, highlights the significance of clastokines in bone remodeling, and explores their potential as treatments for bone diseases suggesting future directions for the field.

## Introduction

Bone serves as a specialized, mineralized connective tissue that provides protection, mechanical support to internal tissues, houses the bone marrow, and permits locomotion (Robling et al., 2006). Despite its seemingly static nature, bone is a highly dynamic organ that undergoes continuous remodeling throughout life. Bone remodeling relies on the coordinated activity of osteoclasts, which resorb old or damaged bone, and osteoblasts, which produce and deposit new bone matrix (Sims and Gooi, 2008; Matsuo and Irie, 2008).

Osteoblasts derive from mesenchymal stem cells (MSCs) and account for 4–6% of the total resident cells in bone (Capulli et al., 2014). Their primary function is the production of bone matrix by secretion of type I collagen and proteoglycans and the subsequent mineralization (Florencio-Silva et al., 2015; Downey and Siegel, 2006). Mature osteoblasts can either undergo apoptosis (Jilka et al., 1998), become embedded in the matrix as osteocytes (Compton and Lee, 2014), or transition into bone lining cells (Miller and Jee, 1987).

Osteoclasts come from a different cell source, originating from mononuclear cells of the hematopoietic lineage (Crockett et al., 2011; Asagiri and Takayanagi, 2007), which upon differentiation and activation form large, multinucleated, highly motile cells that carry out their bone resorption activity. Actively resorbing osteoclasts polarize and their plasma membrane assumes a folded appearance when in contact with bone surface, known as the ruffled border. Within the ruffled border, vacuolar-type H+-ATPase (V-ATPase) and chloride channels contribute to acidifying the extracellular environment, enabling the dissolution of hydroxyapatite crystals (Stenbeck, 2002). This process is further facilitated by enzymes such as tartrate-resistant acid phosphatase (TRAP), cathepsin K (CTSK), and matrix metalloproteinase-9 (MMP-9), which are released into the Howship lacuna (Ljusberg et al., 2005; Mulari et al., 2003). Degradation products are subsequently endocytosed across the ruffled border and transcytosed to the functional exit site on the opposite side of the cell, where they are secreted (Arana-Chavez and Bradaschia-Correa, 2009).

Osteoblast-mediated bone formation and osteoclast-mediated bone resorption are tightly coupled processes, in which both cell types continuously communicate to preserve bone homeostasis. Imbalances in this delicate equilibrium are associated with various bone diseases, including osteoporosis and osteogenesis imperfecta (Seeman, 2003; Etich et al., 2020). While it has long been recognized that osteoblasts can regulate osteoclast differentiation and activity through direct cell–cell communication or by release of soluble factors (Chen et al., 2018; Phan et al., 2004; Boyce, 2013; Martin, 2004), questions regarding how osteoblasts are recruited to the sites of bone remodeling and how they can produce the appropriate amount of bone remained unanswered for a considerable time.

In the last decades, advancements in the understanding of certain cellular events and pathways have shed new light on the osteoclast’s ability to promote and direct bone formation. Such positive correlation between systemic bone resorption and bone formation has been defined as ‘coupling’. The coupling mechanism is necessary to achieve termination of osteoclastic bone resorption and consequent osteoblastic bone formation to fill resorption lacunae with new bone (Sims and Martin, 2014).

Interactions between membrane-bound receptors located on osteoclast and osteoblast surfaces, as well as matrix-derived factors released during bone resorption can drive bone formation to the required sites. More recently, it has become evident that several osteoclast-secreted coupling factors, known as clastokines, also play a role in preserving bone homeostasis by influencing osteoblast differentiation and activity. Techniques such as Alizarin Red staining and evaluation of Alkaline Phosphatase activity in osteoblast cultures treated with osteoclast conditioned media, together with the analysis of bone parameters in osteoclast-specific conditional knockout mouse models, have demonstrated that these factors are able to promote osteoblast differentiation and mineralization *in vitro* and *in vivo*, thereby expanding the known functions of osteoclasts (Drissi and Sanjay, 2016; Cappariello et al., 2014).

This review offers an integrated perspective on the active role of osteoclasts as regulators of bone formation, beyond their conventional role as bone-resorbing cells. We will thoroughly explore the currently known coupling factors and clastokines, discussing their functions, mechanism of activity, and their impact on bone homeostasis. Recognizing how osteoclasts actively collaborate with other members of the bone multicellular unit (BMU) is not only critical to better understand bone homeostasis but also presents an opportunity for the identification of new therapeutic targets for the treatment of bone diseases.

## Matrix-derived signals released during bone resorption

It has long been acknowledged that bone matrix harbors various osteotropic factors that, upon release during osteoclastic bone resorption, can directly or indirectly affect the bone formation activity of osteoblasts. Indeed, growth factors released from the matrix were the first to be identified as coupling factors.

**Figure 1.**
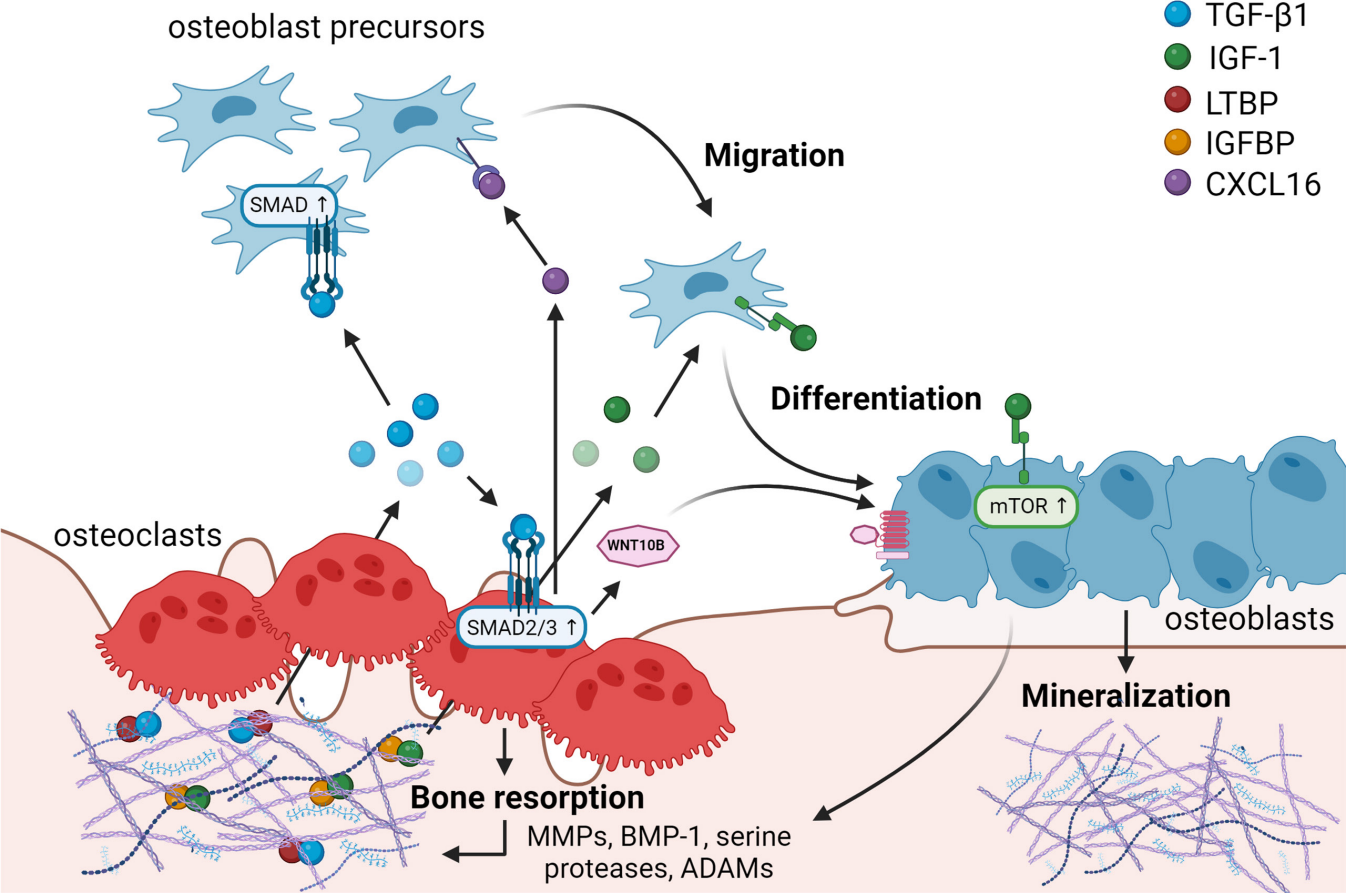
Matrix-derived coupling factors. To carry out their bone resorptive activity, mature osteoclasts secrete serine proteases, MMPs, ADAMs, and BMPs that cleave latency-associated proteins and liberate coupling factors from the extracellular matrix (ECM). TGF-β1 and IGF-1 are the two major factors that are released from the ECM following osteoclast bone resorption. TGF-β1, released after cleavage of LTBP by osteoclast-secreted proteases, acts on osteoblast precursors by activating SMAD signaling to promote cell migration, and on osteoclasts to stimulate the production of WNT10B and CXCL16. WNT10B stimulates osteoblast differentiation and mineralization, while CXCL16 collaborates with TGF-β to enhance osteoblast precursor migration to the resorptive sites. IGF-1 is activated after cleavage of its regulatory protein IGFBP by proteases secreted by osteoblasts upon bone resorption. Active IGF-1 induces differentiation of osteoblast precursors recruited by TGF-β1 by activating the mammalian target of rapamycin(mTOR) signaling pathway. BMP-1: bone morphogenetic protein 1; CXCL16: C-X-C motif chemokine ligand 16; IGF-1: insulin-like growth factor 1; IGFBP: insulin-like growth factor-binding protein; LTBP: latent TGF-β-binding protein; MMPs: metalloproteinases; TGF-β1: transforming growth factor-β1.

A significant factor impacted by bone resorption is latent, matrix-bound transforming growth factor-β1 (TGF-β1), which becomes activated and released by osteoclasts (Figure 1). Latent mature TGF-β is covalently linked to its latency-associated propeptide (LAP) in the matrix. This small complex can further become associated with a latent TGF-β-binding protein (LTBP) (Janssens et al., 2005). Bone resorption by osteoclasts can contribute to creating an acidic environment able to activate TGF-β (Oreffo et al., 1989), but also induces the release of several proteases, such as matrix metalloproteinases (MMPs), Bone morphogenetic protein 1 (BMP-1), and serine proteases that cleave either LAP or LTBP, liberating active TGF-β (Dallas et al., 2002; Ge and Greenspan, 2006).

Active TGF-β1 has been shown to directly induce migration of osteoprogenitors toward bone remodeling sites (Tang et al., 2009; Ota et al., 2013b). In this study, bone MSCs were observed to migrate in response to a TGF-β1 gradient, exhibiting typical lamellipodia-like protrusions, in a process mediated by SMAD signaling (Tang et al., 2009). Besides its direct effects on osteoblasts, TGF-β1 also stimulates osteoclasts to secrete several coupling factors. Notably, TGF-β1-mediated SMAD2/3 signaling stimulates the release of WNT10B by osteoclasts to promote its clastokine activity, as demonstrated by reduced bone formation in response to TGF-β administration in presence of the WNT inhibitor dickkopf-1 (DKK-1) (Ota et al., 2013a). Additionally, TGF-β1 was shown to increase osteoclast expression of the chemokine C-X-C motif chemokine ligand 16 (CXCL16), further enhancing osteoblast migration to recover from bone loss due to bone resorption (Ota et al., 2013b). At the same time, TGF-β1 was also shown to directly stimulate leukemia inhibitory factor (LIF) expression through MEK and SMAD2/3 activation (Ota et al., 2013b). LIF, previously described to act synergistically with TGF-β to promote cell survival (Marzella et al., 1999), opposed direct TGF-β1-mediated stimulation of osteoblast migration (Ota et al., 2013b), pointing out a fine regulation of TGF-β activity in bone metabolism. Taken together, these findings highlight the coupling role of TGF-β in activating osteoblast precursors both directly and through stimulation of several clastokines in osteoclasts during bone resorption.

At bone remodeling sites, the exposed bone matrix creates a stiff and elastic microenvironment capable to drive osteoblast differentiation (Engler et al., 2006). At these locations, other factors are present and can participate in promoting differentiation into osteoblasts.

Insulin-like growth factor 1 (IGF-1), the predominant growth factor in the bone matrix, is stored in the matrix bound to the regulatory protein insulin-like growth factor-binding protein (IGFBP) (Crane and Cao, 2014). IGF-1 can be activated after cleavage of IGFBP by MMPs, BMP-1, and ADAM-9 secreted by osteoblasts upon resorption of bone matrix by osteoclasts. If osteoclasts also release IGFBP proteases is still unclear (Figure 1; Crane and Cao, 2014; Thrailkill et al., 1995; Kim et al., 2011; Mohan et al., 2002). Active IGF-1 induces osteoblastic differentiation of MSCs through the activation of the mTOR signaling pathway (Xian et al., 2012). IGFBP3 regulates IGF-1 lifespan, mediating the incorporation of circulating IGF-1 in the bone matrix to carry out its role during bone remodeling (Xian et al., 2012). Interestingly, *in vivo* studies have revealed a direct correlation between IGF-1 concentration and bone mass, which declines with age (Xian et al., 2012), suggesting a potential role for IGF-1 in the maintenance of bone mass throughout life.

## Regulation of osteoblast differentiation through direct cell–cell interaction with osteoclasts

Osteoblasts are known to regulate osteoclast differentiation by direct cell–cell communication through membrane-bound receptors, for example through the tumor necrosis factor receptor superfamily member 6 (FAS) ligand (FASL)–FAS axis (Wang et al., 2015). However, the capability of osteoclasts to control osteoblast differentiation through direct cell–cell interaction remains understudied. Conventional bone histomorphometric analyses have not been able to show direct binding between these two cell types, resulting in the traditional view of bone remodeling within the BMU as a sequential process, in which bone forming and bone resorptive activities are thought not to occur together but are spatiotemporally distinct from each other (Hattner et al., 1965). However, the advent of cutting-edge technologies, such as intravital two-photon microscopy, has demonstrated that contact areas between osteoblasts and osteoclasts are indeed present and play vital roles in bone homeostasis, even if the mechanisms remain elusive (Furuya et al., 2018). Below we provide examples of recent data describing signaling through direct cell–cell contact between osteoclasts and osteoblasts to change each other’s activity and function. While there is clear evidence for such signaling mechanisms, further research needs to be performed in order to understand their specific spatiotemporal occurrence.

## EFNB2–EPHB4

**Figure 2.**
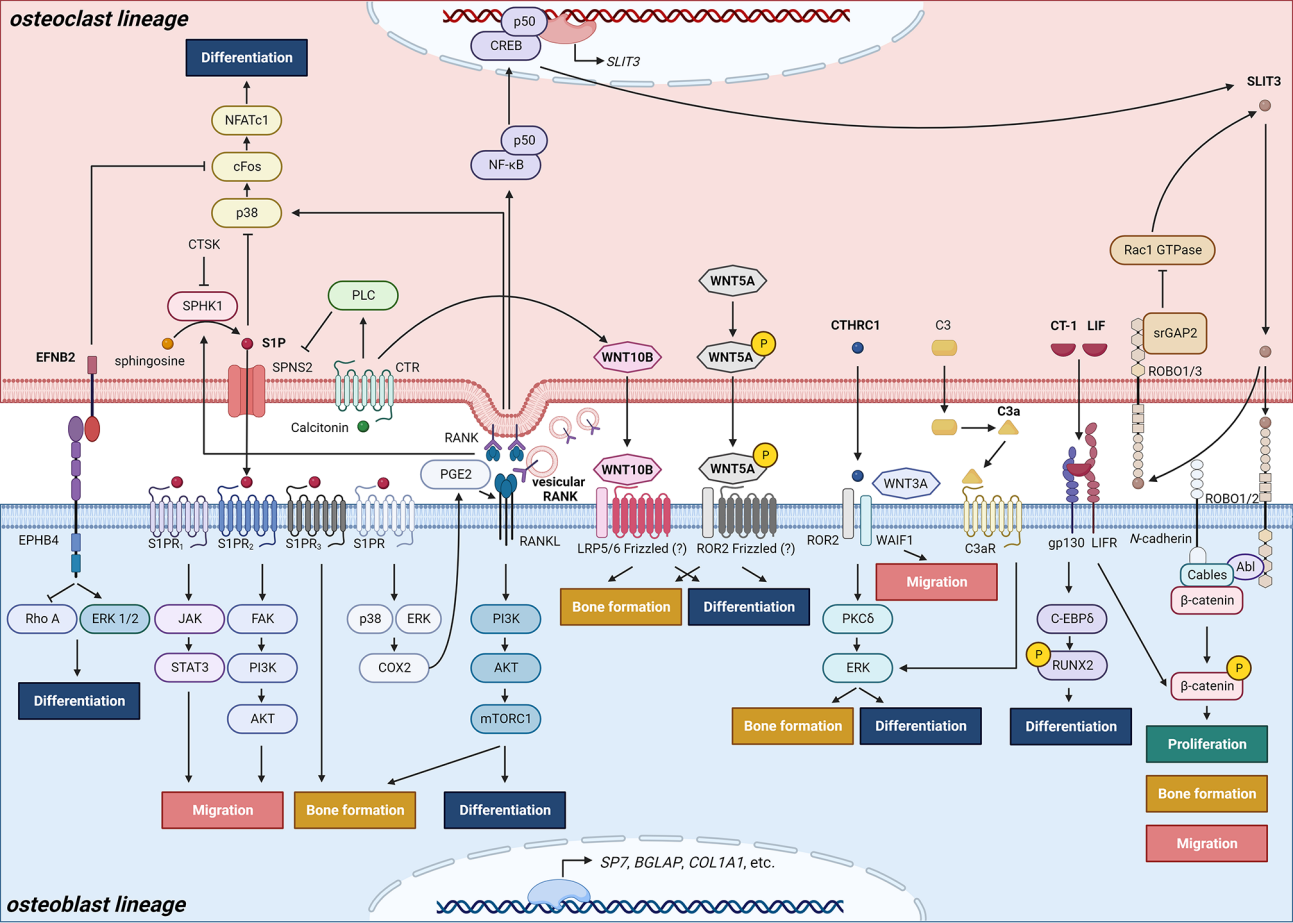
Schematic representation of clastokines produced by osteoclasts and osteoclast precursors and interaction with their receptors on cells from the osteoblast lineage. The picture shows the pathways that were demonstrated to carry out clastokine anabolic function *in vivo* and their known interactions. However, many mechanisms and molecular players remain poorly understood.

Erythropoietin-producing hepatocellular carcinoma tyrosine kinase receptors (EPH) and their membrane-bound ligands, the Ephrins (EFN), play important roles in development and homeostasis, including axon guidance and angiogenesis (Pasquale, 2010). Ephrin B2 (EFNB2) and its receptor EPHB4 are an example of molecules able to mediate osteoclast–osteoblast interaction by simultaneous signal transduction in both cell types, resulting in bidirectional signaling. In reverse signaling (osteoblast to osteoclast), the interaction between EPHB4, located on osteoblasts, and EFNB2 on osteoclasts has been demonstrated to suppress osteoclastogenesis by blocking the c-Fos-NFATc1 cascade (Zhao et al., 2006; Figure 2). Conversely, in forward signaling (osteoclast to osteoblast), EFNB2 activates tyrosine phosphorylation-dependent EPHB4 signaling in osteoblast precursors, promoting osteoblast differentiation and preventing apoptosis, likely through attenuated RhoA activity (Zhao et al., 2006; Tonna et al., 2014). Transgenic mice overexpressing EPHB4, characterized by increased femoral bone density, confirmed this mechanism *in vivo* (Zhao et al., 2006).

## Connexin 43

Conditional deletion of Bone Morphogenetic Protein Receptor 1 (BMPR1) in murine osteoclasts revealed an interesting possible pathway for osteoclast–osteoblast communication requiring direct cell–cell contact. Loss of BMPR1 was shown to result in increased bone formation due to increased levels of its downstream target Connexin 43 (CX43/GJA1), a gap junction protein with a critical role as positive regulator of both osteoblast and osteoclast differentiation (Ilvesaro et al., 2000; Watkins et al., 2011; Ransjö et al., 2003). Gap junctions are important propagators of anabolic signals and agents such as parathyroid hormone (PTH) (Chung et al., 2006). This finding allows to speculate that CX43 permits trafficking of small molecules that act as coupling factors from osteoclasts to osteoblasts and point out BMPR1 as a negative regulator of osteoclast-induced bone formation (Okamoto et al., 2011; Shi et al., 2017), but further studies are required to better understand how the direct cell–cell contact event unfolds.

## SEMA4D–Plexin-B1–ErbB2

Traditionally, the coupling mechanism is associated with the promotion of bone anabolism by osteoclasts. However, osteoclast-derived signals do not always connect bone resorption to bone formation; in some cases, they can separate the two activities.

An inhibitory role has been described for Semaphorin 4D (SEMA4D), a transmembrane protein strongly expressed in osteoclasts but not in osteoblasts (Dacquin et al., 2011; Negishi-Koga et al., 2011). While SEMA4D can be partially secreted as a soluble factor, its membrane-bound form plays a more significant role in bone communication (Negishi-Koga et al., 2011). Likely during the initiation phase of bone remodeling, SEMA4D binds to a receptor complex on osteoblasts consisting of Plexin-B1 and erythroblastic leukemia viral oncogene homolog 2 (ErbB2). The interaction leads to the activation and autophosphorylation of ErbB2, which in turn phosphorylates Plexin-B1. The subsequent activation of RhoA–Rho-associated protein kinase (ROCK) pathway leads to reduced tyrosine phosphorylation of insulin receptor substrate 1 (IRS-1), a positive regulator of osteogenesis (Wang et al., 2018; Xi et al., 2019), thus resulting in inhibited bone formation (Negishi-Koga et al., 2011). SEMA4D signaling also promotes osteoblast motility away from the sites of resorption by decreasing cadherin-11 expression (Negishi-Koga et al., 2011). Due to its inhibitory role, SEMA4D has been suggested not to be classified as a coupling factor, but rather as a bone communication factor important for intercellular crosstalk during bone remodeling (Negishi-Koga and Takayanagi, 2012).

## Osteoclast-secreted coupling factors (clastokines)

The concept that osteoclasts might secrete factors promoting osteoblast differentiation is not recent. Scientists began speculating about the existence of osteoclast-derived coupling factors as early as the 1980s (Farley et al., 1987), with more focused efforts initiated in the early 2000s (Martin and Sims, 2005; Karsdal et al., 2008; Karsdal et al., 2007). In 2008, the discovery that conditioned media from human osteoclasts was able to induce bone nodule formation by osteoblasts in a dose-dependent manner sparked further investigation into the mechanisms underlying osteoclastic control of osteoblasts (Karsdal et al., 2008). To this effect, molecules secreted by osteoclasts that demonstrated the ability to influence osteoblastic activity or function have widely been termed ‘clastokines’, and their roles have started to be explored both *in vitro* and *in vivo* (Figure 2, Table 1).

**Table 1.** Summary of coupling factors linking bone resorption to bone anabolism.

*Known coupling factor*		*Osteoblast receptor*	*Reported coupling and anabolic effects*	*Mechanisms*	*References*
***Membrane bound***					
**EFNB2**	Ephrin B2	EPHB4	Promotes osteoblast differentiation *in vitro* and *in vivo*. Prevents osteoblast apoptosis *in vitro* and *in vivo*.	Ephrin B2 binds to EPHB4 receptor and inhibits RhoA activity.	Zhao et al., 2006; Tonna et al., 2014
***Matrix derived***					
**TGF-β1**	Transforming growth factor-β1	TGFβR1	Promotes MSCs migration to the remodeling sites *in vitro* and *in vivo*.	SMAD signaling mediates the creation of a TGF-β1 gradient that directs cell migration.	Tang et al., 2009; Ota et al., 2013b; Ota et al., 2013a
			Stimulates the secretions of other clastokines such as WNT10B, LIF, and CXCL16.	TGF-β activates SMAD2/3 signaling to stimulate CXCL16 expression and SMAD2/3 and MEK signaling to stimulate LIF expression.	
**IGF-1**	Insulin-like growth factor 1	IGF1R	Promotes osteoblast differentiation.	IGF-1 activates mTOR1 signaling pathway.	Xian et al., 2012
***Secreted (clastokines)***					
**S1P**	Sphingosine-1-phosphate	S1Ps receptors	Promotes osteoblast differentiation *in vitro*. Promotes bone formation *in vivo*.	S1P is phosphorylated by SPHK1, an event inhibited in presence of cathepsin K. S1P is secreted from the osteoclasts thanks to SPNS2 export protein. In the extracellular space, S1P binds its S1PR_3_ receptor located on osteoblast surface. In presence of calcitonin, binding its receptor (CTR) on osteoclast membrane, PLC signaling results in the inhibition of SPNS2 expression.	Ryu et al., 2006; Quint et al., 2013; Pederson et al., 2008; Keller et al., 2014; Lotinun et al., 2013
			Enhances osteoblast migration and survival *in vitro*.	MSCs chemokinesis is stimulated by JAK/STAT3 and FAK/PI3K/AKT signaling pathways via S1PR_1_ and S1PR_2_, respectively.	
			Increases RANKL expression *in vitro*.	S1P binding to its receptor leads to the activation of ERK and p38 to upregulate COX2 level, leading to increased PGE2 production in the osteoblast.	
			Suppresses osteoclast differentiation *in vitro*.	Following RANKL-binding RANK on osteoclast precursors, the downstream activation of p38, c-Fos, and NFATc1 stimulates osteoclast differentiation. RANKL also stimulates S1P production by SPHK1. Intracellular S1P constitutes a negative feedback loop by suppressing p38 and inhibiting osteoclast differentiation.	
**WNT10B**	Wingless-type MMTV integration site family, member 10B	N/A	Promotes osteoblast differentiation *in vitro*. Improves bone mineralization, bone quality and quantity *in vivo*.	WNT10B expression and secretion is stimulated by calcitonin and by TGF-β1.	Ota et al., 2013a; Pederson et al., 2008; Zheng et al., 2019; Hsiao et al., 2020
**C3a**	Complement component 3a	C3aR	Promotes osteoblast differentiation *in vitro*. Stimulates bone formation and maintains bone mass and structure.	C3a binds to its osteoblast receptor C3aR, and then likely induces ERK pathway, but the mechanism is still poorly understood.	Matsuoka et al., 2014
**CTHRC1**	Collagen Triple Helix Repeat Containing 1	WAIF1, ROR2	Promotes osteoblast differentiation and mineralization *in vitro*. Suppresses adipocytic differentiation *in vitro*. Maintains bone mass and trabecular structure through regulation of bone formation *in vivo*.	WAIF1 and ROR2 receptors, located on the surface of stromal cells, bind CTHRC1 and activate the PKCδ/ERK pathway, stimulating differentiation into osteoblasts.	Kimura et al., 2008; Takeshita et al., 2013; Matsuoka et al., 2018
			Stimulates chemotaxis of stromal cells.	The stimulation occurs in presence of WNT3A, supporting a crosstalk between CTHRC1 and WNT pathway.	
**WNT5A**	Wingless-type MMTV integration site family, member 5A	N/A	Maintains bone mass through regulation of bone formation *in vivo*.	WNT5A derived from osteoclasts is phosphorylated at Serine residues to acquire its function.	Roberts et al., 2020
**SLIT3**	Slit Guidance Ligand 3	ROBO1/2	Stimulates osteoblast migration and proliferation *in vitro* and *in vivo*. Promotes bone formation *in vivo*.	During osteoclast differentiation, RANKL induces the activation of NF-κB p50 and CREB, increasing SLIT3 expression. SLIT3 signaling is mediated by ROBO1 and ROBO2 receptors in osteoblasts. SLIT3/ROBO complex associates with Abl kinase, *N*-cadherin, and β-catenin. Following Abl phosphorylation, *N*-cadherin-associated β-catenin is released, promoting osteoblast migration and proliferation.	Kim et al., 2018; Shin et al., 2020
			Suppresses bone resorption in an autocrine manner *in vivo*.	SLIT3 signaling is mediated by ROBO1 and ROBO3 receptors in osteoclasts. Osteoclast SrGAP2 intracellularly binds the SLIT3/ROBO complex and inhibits Rac1 GTPase, resulting in the inhibition of TNF-α-mediated osteoclastogenesis. The pathway has negative feedback: Rac1 promotes the production of SLIT3, which recruits SrGAP2 to inhibit Rac1 expression.	
**CT-1**	Cardiotrophin-1	LIFR, gp130	Increases osteoblast activity *in vitro*. Suppresses adipocytic differentiation *in vitro*. Promotes bone formation *in vivo*. Inhibits sclerostin production.	CT-1 induces the expression of C/EBPδ, which acts in synergy with RUNX2 to promote osteocalcin expression through a C/EBP enhancer element. CT-1 induces high level of STAT3 and ERK phosphorylation *in vitro*. CT-1 signals through LIFR and gp130 receptors on osteoblast membrane. A third, CT-1-specific receptor subunit that has still not yet been identified might also be involved.	Sims and Walsh, 2010; Walker et al., 2008; Walker et al., 2010
**LIF**	Leukemia inhibitory factor	LIFR, gp130	Promotes bone formation *in vivo*.	LIF expression and secretion are stimulated by TGF-β1 through SMAD2/3 signaling. LIF binds LIFR and gp130 receptors on osteoblast surface. LIF signaling downregulates sclerostin expression and upregulates WNT/β-catenin pathway in osteoblasts.	Ota et al., 2013b; Sims and Walsh, 2010; Walker et al., 2010
***Putative clastokines (limited data)***					
**BMP6**	Bone morphogenetic protein 6	N/A	Promotes MSCs differentiation, mineralization, and migration *in vitro*.	N/A	Pederson et al., 2008
**Afamin**	Afamin	N/A	Induces osteoblast precursors migration *in vitro* and *in vivo*.	Afamin activates the AKT pathway in osteoblasts.	Kim et al., 2012
**PDGF-BB**	Platelet-derived growth factor BB	PDGFR-β	Induces a migratory response in MSCs *in vitro*. Inhibits osteoblast differentiation *in vitro*. Promotes bone formation in vivo*.	N/A	Kreja et al., 2010; Sanchez-Fernandez et al., 2008; Kubota et al., 2002; Nash et al., 1994; Mitlak et al., 1996
**HGF**	Hepatocyte growth factor	cMET	Promotes osteoblast proliferation *in vitro*. Stimulates osteoclast migration *in vitro*.	HGF binding to cMET activates the PI3K, Akt, and c-Src cascade, which promotes the binding of c-Jun to the AP-1 site. This results in the stimulation of OPN expression in osteoblasts.	Grano et al., 1996; Chen et al., 2012
**CXCL16**	Chemokine (C-X-C motif) Ligand 16	N/A	Stimulates osteoblast migration *in vitro*.	CLXC16 expression and secretion are stimulated by TGF-β1 through SMAD2/3 signaling.	Ota et al., 2013b
***EV-contained***					
**Vesicular RANK**	Receptor activator of nuclear factor κB	RANKL	Promotes osteoblast differentiation and bone formation *in vitro* and *in vivo*.	The binding activates RANKL reverse signaling, triggering, the PI3K–Akt–mTORC1 pathway with subsequent production of RUNX2 by osteoblasts.	Ikebuchi et al., 2018
**miR-214-3**p		EPHA2	Suppresses osteoblast differentiation and bone formation *in vitro* and *in vivo*.	miR-214-3p inhibits ATF4 and Osterix expression.	Li et al., 2016; Wang et al., 2013; Shi et al., 2013
**miR-324**		N/A	Enhances osteoblast differentiation and mineralization *in vitro* and *in vivo*.	miR-324 downregulates the expression of ARHGAP1, a member of the RhoGAP family containing GTPase-activating proteins, stimulating RhoA/ROCK signaling.	Liang et al., 2021

MSCs: mesenchymal stem cells; N/A: unclear or missing evidence. *Still controversial.

## Sphingosine-1-phosphate

Indeed, Sphingosine-1-phosphate (S1P) was one of the initially identified clastokines. S1P is a bioactive lysosphingolipid playing fundamental roles in morphogenesis, including tissue induction, collective cell migration, and biomechanical signaling (Mendelson et al., 2014). It is produced by osteoclasts through sphingosine phosphorylation by Sphingosine kinase 1 (SPHK1) and exerts its functions by interacting with its receptors located on the membrane of several cell types (Figure 2). S1P importance for bone homeostasis comes not only from its capability to control migration and behavior of osteoclast precursors (Ishii et al., 2009), but also from its uncovered role as a clastokine.

During osteoclast differentiation, increased SPHK1 expression and activity lead to high S1P levels (Ryu et al., 2006). S1P is then released from osteoclasts to the extracellular space through the transmembrane transporter SPNS2. S1P was shown to promote migration of MSCs *in vitro* through activation of the JAK/STAT3 and FAK/PI3K/AKT signaling pathways mediated by its receptors S1PR_1_ and S1PR_2_, respectively (Quint et al., 2013). Moreover, S1P enhances bone formation *in vivo* by binding to its osteoblast receptor S1PR_3_ (Pederson et al., 2008; Keller et al., 2014). In a different *in vitro* study, S1P signaling activated extracellular-signal regulated kinases (ERK) and p38, which upregulated cyclooxygenase-2 (COX2) levels, resulting in increased prostaglandin E_2_ (PGE2) production and subsequent enhanced Receptor Activator Of Nuclear Factor Kappa B Ligand (RANKL) expression in osteoblasts (Ryu et al., 2006). S1P production is finely regulated through multiple mechanisms. Cathepsin K has been shown to inhibit S1P production by repressing SPHK1 kinase activity (Lotinun et al., 2013). Similarly, the thyroid hormone calcitonin, which binds the transmembrane calcitonin receptor (CTR) on the osteoclast membrane, acts as a negative regulator of SPNS2 expression likely through phospholipase C signaling thereby reducing S1P secretion (Keller et al., 2014). Thus, CTR-deficient mice exhibit higher S1P levels in bone, resulting in an increased bone formation rate (Keller et al., 2014).

Administration of an S1P analog able to act on four out of the five S1P receptors resulted in increased bone formation and mineralization in mice (Weske et al., 2018). However, increased OPG levels and reduced osteoclast numbers were observed in treated animals, leading to questioning if the positive effect is due to S1P anabolic activity or mostly to its autocrine inhibitory effect on osteoclasts (Weske et al., 2018; Sims and Martin, 2020). Further studies will help to clarify the primary role of S1P on bone metabolism *in vivo*.

## Collagen triple helix repeat containing 1

Collagen triple helix repeat containing 1 (CTHRC1) is a highly conserved secreted glycoprotein that was first identified in the arterial wall in response to injury (Pyagay et al., 2005; LeClair and Lindner, 2007), where it acted as a regulator of TGF-β1 signaling during vascular remodeling (LeClair and Lindner, 2007). Later, it was found to be expressed in the cartilage growth plate during mouse development (Kimura et al., 2008). In adulthood, CTHRC1 is primarily found in bone, with minimal expression in the brain (Takeshita et al., 2013). CTHRC1 was shown to stimulate osteogenic differentiation and suppression of adipocytic differentiation of marrow stromal cells (Takeshita et al., 2013). The importance of CTHRC1 in promoting bone formation to preserve bone mass and trabecular structure was ascertained through conditional deletion in Ctsk positive cells and global deletion experiments which resulted in low bone mass characterized by decreased trabecular number and thickness in mice (Takeshita et al., 2013).

Interestingly, CTHRC1 expression has been demonstrated to strongly correlate with the activity of osteoclasts, as demonstrated by its increased expression in osteoclasts placed on dentin or hydroxyapatite, and reduced expression after alendronate treatment or aging (Takeshita et al., 2013). Its association with high bone turnover states suggests its potential as a biomarker or therapeutic target for the diagnosis and treatment of bone diseases such as osteoporosis. WNT-activated inhibitory factor 1 (WAIF1), encoded by the *trophoblast glycoprotein* (*TPBG*) gene, was identified as a receptor for CTHRC1 on stromal cells and osteoblasts (Matsuoka et al., 2018), and WAIF1 binding to CTHRC1 and Tyrosine-Protein Kinase Transmembrane Receptor ROR2 was shown to promote osteoblast differentiation through activation of the PKCδ–ERK pathway (Matsuoka et al., 2018; Figure 2). Additionally, osteoblast-specific deletion of WAIF1 resulted in reduced bone formation, similar to the effects observed with conditional CTHRC1 knockout in osteoclasts, confirming the dysregulation of the coupling mechanism mediated by both proteins (Matsuoka et al., 2018).

While the *in vivo* data unequivocally show the role of CTHRC1 as a promoter of osteoblast differentiation, its actual production by osteoclasts has recently become conflicting. While an earlier study showed *Cthrc1* transcript expression in multinucleated osteoclasts on mouse trabecular bone, latest research has demonstrated that, in the same tissue, CTHCR1 protein does not colocalize with the osteoclast marker TRAP and is rather present in osteoblasts and osteocytes (Jin et al., 2017). More studies are required to clarify this discrepancy and demonstrate whether CTHRC1 truly acts as a coupling factor secreted by osteoclasts or signals within the osteoblast lineage.

## Complement component C3a

C3a is a small peptide produced through the proteolytic cleavage of C3 during the initial phases of complement activation (Dunkelberger and Song, 2010) and is expressed by several cell types, such as immune cells (Lubbers et al., 2017), hepatocytes (Han and Zhang, 2021), but also osteoblasts and osteoclasts (Ignatius et al., 2011). C3a was identified in conditioned media of osteoclasts as a factor promoting osteoblast differentiation *in vitro*. Its role as a clastokine was further elucidated after observing reduced osteoblast mineralization following knockdown of *C3* gene expression in osteoclasts and increased ALP activity following administration of a C3a receptor (C3aR) agonist in osteoblast cultures (Matsuoka et al., 2014). Moreover, increased levels of C3a were reported in ovariectomized mice, characterized by a high turnover state (Matsuoka et al., 2014). In this model, administration of a C3aR antagonist inhibited bone formation and exacerbated bone loss and trabecular architecture deterioration, providing evidence of C3a function *in vivo* (Matsuoka et al., 2014). The downstream signaling pathways that are activated through osteoclast-derived C3a interaction with C3aR on osteoblasts likely involve ERKs, but the precise mechanism remains to be determined (Matsuoka et al., 2014; Figure 2). Notably, C3 expression was observed to increase in osteoblasts treated with 1α,25-dihydroxyvitamin D3 (Matsuoka et al., 2014; Sato et al., 1991); however, C3a protein could not be detected in osteoblast conditioned medium (Sato et al., 1991). This suggests the presence of a specific processing system for C3a production present in osteoclasts but not in osteoblasts (Sato et al., 1991), which may ultimately influence its function depending on the cell source.

## Wingless-type MMTV integration site family, member 5A

The WNT signaling pathway has great relevance in the skeletal field, as WNT proteins are well-known regulators of skeletal development and homeostasis (Zhong et al., 2014). They carry out their function through two pathways: the canonical WNT-β–catenin pathway and the noncanonical WNT pathway, that is further classified into two sub-pathways, the WNT–Ca^2+^ and the WNT–JNK pathways (Albrecht et al., 2021; Lojk and Marc, 2021).

Wingless-type MMTV integration site family, member 5 (WNT5A) secreted by osteoblasts was found to enhance osteoclastogenesis *in vivo* following RANKL stimulation (Maeda et al., 2012). Osteoblast-derived WNT5A was shown to interact with ROR2 located on the osteoclast surface, inducing noncanonical WNT signaling. This leads to the phosphorylation of JNK, followed by c-Jun-Sp1-mediated transactivation of the *Tnfrsf11a* gene, encoding the receptor RANK, which in turn results in increased RANK expression in osteoclast precursors (Maeda et al., 2012). Hence, the WNT5A–ROR2 axis promotes osteoclastogenesis, controlling osteoclast number, and bone resorption activity through coordinated signaling (Maeda et al., 2012). Additionally, WNT5A can promote osteoclastogenesis by abrogating the inhibitory signals of WNT16, but the mechanism is independent from ROR2 as WNT16 was demonstrated to not be able to bind the receptor (Kobayashi et al., 2015).

Due to the various roles of WNT5A, not only related to osteoblast–osteoclast communication (Gofflot et al., 1998), the hypothesis that WNT5A functionality could be dependent on the cell of origin and cell context was already postulated around 10 years ago (Zhong et al., 2014). A recent study highlighted the complexity of WNT5A mechanisms in preserving bone homeostasis. Starting from the already known ability of osteoblast-derived WNT5A to influence osteoclasts, the study was aimed at investigating the role of osteoclast-derived WNT5A. Conditional removal of WNT5A in osteoclasts resulted in decreased bone mass *in vivo*, which was not due to altered osteoclast number or activity, but due to decreased bone formation by osteoblasts (Roberts et al., 2020). Interestingly, osteoclast-derived WNT5A was shown to differ from osteoblast-derived WNT5A by the presence of post-translational modifications, specifically the phosphorylation of specific Serine residues (Roberts et al., 2020; Figure 2). These findings uncovered an unexpected clastokine role for WNT5A, which is post-translationally modified in a cell-type-specific manner to acquire its function (Roberts et al., 2020). Further studies are needed to define how osteoclast-derived WNT5A communicates with osteoblasts and its mechanism of action.

## WNT10B

WNT10B is another member of the WNT ligand gene family known to specifically activate canonical WNT/β-catenin signaling (Wend et al., 2012). Pederson et al. showed that WNT10B along with S1P was also expressed in osteoclasts and was able to induce human MSC differentiation and mineralization *in vitro* (Pederson et al., 2008).

Recently, researchers have suggested that Cinacalcet, a calcimimetic compound, can improve bone mineralization, bone quantity, and quality in chronic kidney disease mice by increasing WNT10B secretion by osteoclasts (Zheng et al., 2019). Similarly, expression of WNT10B by osteoclasts was also stimulated by calcitonin (Figure 2), resulting in improved bone parameters and increased bone mineralization in ovariectomy-induced osteoporotic rats (Hsiao et al., 2020). These studies suggest that calcitonin may play a role in the coupling mechanism by modulating osteoblast mineralization through the controlled secretion of WNT10B and S1P.

## Leukemia inhibitory factor

The glycoprotein 130 (gp130)-binding cytokine LIF was already known to promote mitogenic activity in osteoblasts as early as 1997 (Cornish et al., 1997). However, its identification as a coupling factor produced by osteoclasts in response to TGF-β1 stimulation was only made recently (Ota et al., 2013b). LIF exerts its clastokine activity by downregulating sclerostin expression and upregulating WNT/β–catenin pathway components in osteoblasts to promote bone formation (Ota et al., 2013b; Figure 2). A recent study on bone biopsies from post-menopausal women revealed LIF to be highly expressed in osteoclasts compared to osteoblasts and to be suppressed by Denosumab treatment, confirming its role as a clastokine to be conserved and present during human bone remodeling (Weivoda et al., 2020).

## Cardiotrophin-1

Cardiotrophin-1 (CT-1) is a member of the IL-6 family of cytokines and was originally discovered as a factor inducing cardiac myocyte hypertrophy (Pennica et al., 1995). While its hypertrophic and cardioprotective effects have been the most extensively characterized, other effects of CT-1 on different tissues have also been reported (Peters et al., 1995; López-Yoldi et al., 2015). CT-1 is expressed in bone, specifically in osteoclasts but not osteoblasts or osteocytes (Sims and Walsh, 2010). Osteoclast-derived CT-1 was described to act as a clastokine to promote osteoblast mineralization *in vitro* and *in vivo* when administered over the calvariae of young mice (Walker et al., 2008). CT-1 knockout in young mice resulted in a significant reduction in trabecular bone volume, trabecular number, and osteoblast number, confirming its role in promoting bone formation, especially during early developmental stages (Walker et al., 2008). CT-1 also appears to regulate, likely through an autocrine mechanism, osteoclast number, size, and function since genetic deletion of CT-1 leads to an increased number of larger osteoclasts and impaired bone resorption (Walker et al., 2008). The mechanism by which CT-1 exerts its effects has been proposed to be related to the activation of CCAAT/enhancer-binding protein δ (C/EBPδ), which induces RUNX2-dependent osteocalcin transcription in osteoblasts (Walker et al., 2008; Figure 2). Increased levels of phosphorylated STAT3 and ERK were also detected after CT-1 administration to stromal cells, indicating their contribution to its clastokine activity (Walker et al., 2008). Similar to LIF, CT-1 is a gp130-binding cytokine. Both their stimulatory signals are mediated by the formation of ligand–receptor complexes with LIF receptor (LIFR) and gp130 heterodimers on the osteoblast membrane (Sims and Walsh, 2010). The gp130 pathway has important key roles in bone homeostasis by regulating the differentiation and activity of osteoblasts, osteoclasts, and chondrocytes (Johnson et al., 2014; Johnson et al., 2015; Liu et al., 2022). The ability of gp130 to mediate clastokine signals sheds new light on the multifaceted activity of this receptor, confirming its fundamental function in preserving bone balance.

## Slit guidance ligand 3

Slit Guidance Ligand 3 (SLIT3) belongs to the SLIT family, a group of secreted proteins acting as molecular cues to control migration in several cell types (Geutskens et al., 2010; Zhang et al., 2009; Dou et al., 2018; Kramer et al., 2001; Wang et al., 1999). SLIT3 involvement in bone homeostasis was only recently acknowledged after its identification in purified conditioned media from mature osteoclasts which was able to stimulate migration of osteoblast lineage cells (Kim et al., 2012). This ability was also confirmed *in vivo* by increased bone marrow mobilization following injection of SLIT3 of GFP-labeled osteoblasts in the mouse tibia (Kim et al., 2018). Two independent groups showed that global knockout of SLIT3 in mice results in reduced skeletal size and a severely osteoporotic phenotype compared to their WT littermates (Kim et al., 2018; Xu et al., 2018). In the first study *Slit3^−/−^* mice also displayed increased bone resorption parameters and osteoclast numbers (Kim et al., 2018), pointing out SLIT3 as an osteoprotective molecule capable of stimulating bone formation while simultaneously suppressing bone resorption. However, those findings were not recapitulated in the study from the second group (Takeshita et al., 2013; Li et al., 2020). Furthermore, while both groups used Ctsk:Cre to create a conditional knockout for SLIT3, only one of them was able to observe an osteoporotic phenotype associated with lack of SLIT3 in osteoclasts (Kim et al., 2018; Li et al., 2020). The different nature of the floxed mice used to carry out the studies could possibly explain the discrepancy (Kim et al., 2018; Xu et al., 2018; Li et al., 2020).

The mechanism by which SLIT3 exerts its function has also been elucidated. SLIT3 is expressed by mature osteoclasts following RANKL stimulation (Kim et al., 2018). After secretion, SLIT3 can bind to Roundabout Guidance Receptors ROBO1 and ROBO2 located on the osteoblast surface, and to ROBO1 and ROBO3 receptors on osteoclasts. In osteoblasts, the downstream SLIT3/ROBO signaling involves the formation of a multimolecular complex consisting of ROBO protein, Abelson (Abl) tyrosine kinase, adaptor protein Cables (Cdk5 and Abl enzyme substrate 1), *N*-cadherin, and β-catenin (Blockus and Chédotal, 2016; Rhee et al., 2007). Following Abl phosphorylation, *N*-cadherin-associated β-catenin is released, leading to the loss of *N*-cadherin-mediated cell adhesion (Kim et al., 2018; Rhee et al., 2007; Koohini et al., 2019), and to β-catenin translocation to the nucleus (Kim et al., 2018), ultimately resulting in increased osteoblast migration and proliferation (Kim et al., 2018; Figure 2). The autocrine signaling of SLIT3 in osteoclasts involves the recruitment of SLIT3–ROBO GTPase activating protein 2 (srGAP2) to the intracellular domain of ROBO1 receptor with consequent Rac1 GTPase inhibition, reduced NF-κB expression and reduced osteoclast differentiation (Shin et al., 2020). A negative feedback loop further regulates the pathway, as Rac1 promotes SLIT3 expression, ultimately leading to increased srGAP2 and Rac1 suppression (Shin et al., 2020; Figure 2).

A recent study from the first group again confirmed SLIT3 increasing expression during osteoclast differentiation and enhanced bone loss in the *Slit3^−/−^* mouse model (Kim et al., 2023), but the authors did not address the ongoing controversy. Multiple pieces of evidence support the role of SLIT3 in the coupling between bone resorption and bone formation (Kim et al., 2018; Shin et al., 2020). A third independent report would likely help to overcome these inconsistencies and clarify SLIT3 relevance as an osteoclast-derived clastokine.

## Putative clastokines

Some factors are currently mentioned in the literature as ‘putative clastokines’ as only limited data are currently available for these molecules, mostly restricted to *in vitro* studies. Therefore, further studies are required to prove their function as clastokines.

## Afamin

The new member of the albumin family, afamin, was first identified in osteoclast conditioned media (Kim et al., 2012). Recombinant afamin was shown to stimulate mouse calvarial osteoblast migration in a dose-dependent manner by inducing lamellipodia formation, suggesting a role as a chemoattractant (Kim et al., 2012). Further confirmation came from an *in vivo* migration experiment in which preosteoblasts, injected together with afamin in mouse tibia, mainly localized in the areas of bone formation (Kim et al., 2012). Afamin was shown to activate AKT signaling *in vitro*, identifying this pathway as a possible mediator of its chemotactic activity (Kim et al., 2012).

## C-X-C motif chemokine ligand 16

The chemokine CXCL16 is a transmembrane protein which is released from the cell membrane following cleavage by disintegrin and metalloproteinase domain-containing protein 10 (ADAM-10) (Abel et al., 2004). Soluble CXCL16 produced by macrophages is responsible for the recruitment of T cells to sustain inflammation in rheumatoid arthritis (van der Voort et al., 2005). A study demonstrated that, in a similar fashion, CXCL16 produced by osteoclasts following TGF-β1 stimulation is able to initiate MSC migration to the sites of bone resorption (Ota et al., 2013b), suggesting that this molecule may act as a clastokine during bone remodeling.

## Bone morphogenetic protein 6

Bone morphogenetic proteins (BMPs) are a large family of growth factors that play critical roles in skeletal development and maintenance. Among the different types of BMPs present in bone, BMP2, BMP4, BMP6, and BMP7 have been shown to be able to induce bone formation *in vivo* (Katagiri and Watabe, 2016). Interestingly, they all can also be expressed by osteoclasts (Spector et al., 2001; Jensen et al., 2010). In particular, BMP6 was identified as an osteoclast-derived factor able to promote human MSC differentiation and mineralization *in vitro*. Furthermore, BMP6 was also reported to promote MSC migration in a wound healing assay (Pederson et al., 2008). No other studies have confirmed the relevance of BMP6 as a clastokine so far, therefore its function in bone homeostasis remains largely uncharacterized.

## Platelet-derived growth factor-BB

Several reports indicate platelet-derived growth factor-BB (PDGF-BB), one of the most potent known chemoattractants for human MSCs (Fiedler et al., 2004), as a functional candidate in the osteoclast-dependent regulation of bone formation. Earlier studies have shown that PDGF-BB can be released from its heparin and collagen matrix bound form during bone resorption (Ross et al., 1986; Sun et al., 2009). However, more recent research has demonstrated that PDGF-BB can also directly be secreted by osteoclasts. Interaction of PDGF-BB with PDGF-receptor β on the osteoblast surface induces the migration of osteoblasts and osteoblast precursors (Kreja et al., 2010), but the downstream cascade of signaling events is still unclear (Jaganathan et al., 2007; Kallin et al., 2004; Rivera et al., 2006). This was confirmed by a significantly reduced chemotactic response of osteoblasts and preosteoblastic cells to osteoclasts in which PDGF-BB expression was downregulated by siRNA (Kreja et al., 2010; Sanchez-Fernandez et al., 2008). One of these works claimed osteoclast chemotactic potency to be dependent on their level of differentiation (Sanchez-Fernandez et al., 2008), but it was demonstrated that the migration stimulatory effect on osteoblasts was not linked to osteoclast resorptive activity as conditioned media from non-resorbing, mononuclear osteoclast cultures were shown to be able to induce osteogenic differentiation (Kreja et al., 2010). This suggests that the ability of osteoclasts to secrete clastokines is not necessarily related to their resorptive state.

Despite the evident role on osteoblast migration, the effect of PDGF-BB on bone formation is still controversial. Some published evidence supports its inhibitory role on osteoblastogenesis *in vitro* (Kubota et al., 2002; O’Sullivan et al., 2007; Fierro et al., 2007), supporting the idea that PDGF-BB increases osteoblast proliferation at the expenses of differentiation. However, some older literature pointed out a positive effect of PDGF-BB on bone formation and bone healing *in vivo*, as its administration accelerated fracture healing in tibial osteotomies in rabbits and increased bone parameters in ovariectomized rats (Nash et al., 1994; Mitlak et al., 1996). Further studies are needed to clarify the mechanisms and effects by which PDGF-BB operates to maintain bone homeostasis *in vivo*. Conditional inactivation of PDGF-BB in osteoclasts or PDGF-β in osteoblasts, along with the administration of osteoclast-derived PDGF-BB in murine models, could represent strategies to address the physiological role of this factor.

## Hepatocyte growth factor

Hepatocyte growth factor (HGF), expressed in both osteoblasts and osteoclasts, was postulated as a coupling factor for the first time in 1996. In this study, HGF produced by osteoclasts was found to increase the number of osteoclasts and, at the same time, to induce proliferation of osteoblasts *in vitro* (Grano et al., 1996). Researchers hypothesized that osteoclasts could act as coordinators of bone formation and bone resorption to maintain bone balance by secreting HGF acting through autocrine and paracrine mechanisms (Grano et al., 1996). HGF stimulates osteoblasts by binding to its canonical receptor c-Met on the osteoblast surface. The consequent activation of PI3K, AKT, and c-Src leads to enhanced binding of c-Jun to the AP-1 site and increased expression of osteopontin (OPN) (Chen et al., 2012).

## Osteoclast-secreted extracellular vesicles

The coupling of bone resorption to bone formation involves not only the secretion of factors at the remodeling site, but also the production of specific extracellular vesicles (EVs) by osteoclasts. EVs serve as important messengers in the bone microenvironment, delivering cytokines, microRNAs (miRNAs), and growth factors from one cell type to another (Liu et al., 2018). Unlike paracrine signaling, EVs provide a protective environment for their cargo, ensuring effective communication, and fine regulation of the crosstalk between bone cells (Liu et al., 2018).

miRNAs have emerged as important regulators of bone homeostasis, although whether they were secreted or shuttled through EVs remained uncertain for some time (Collino et al., 2010; Lian et al., 2012). A recent study identified 12 miRNAs inside osteoclast-derived EVs, among which miR-214-3p was found to be delivered to osteoblasts, acting as an intercellular messenger (Li et al., 2016). miR-214-3p targets Activating Transcription Factor 4 (ATF4), a transcription factor involved in osteoblast differentiation, thereby suppressing osteoblast differentiation and subsequent bone formation (Li et al., 2016; Wang et al., 2013). Previous work did also show miR-214-3p-mediated suppression of osteogenic differentiation of C2C12 myoblasts by targeting Osterix (Shi et al., 2013). The proposed mechanism for miR-214-3p delivery to osteoblasts involves the recognition of EFNA2, located on the vesicle membrane, by its receptor EPHA2 on osteoblasts, resulting in increased RhoA activity (Sun et al., 2016). Another miRNA, miR-324, present in osteoclast-derived EVs, was shown to be able to promote osteogenic differentiation *in vitro* and to induce bone formation in a calvaria defect model *in vivo* (Liang et al., 2021). miR-324 coupling activity is carried out by downregulation of the expression of ARHGAP1, a member of the RhoGAP family containing GTPase-activating proteins, stimulating RhoA/ROCK signaling, and ultimately enhancing osteoblast differentiation and mineralization (Liang et al., 2021). These data prove that miRNAs produced by osteoclasts can influence osteoblast behavior during bone remodeling, acting as intercellular communication tools to regulate bone homeostasis.

The established communication between osteoclasts and osteoblasts through the RANKL–RANK–OPG axis has been a hallmark of osteoblastic control of osteoclasts (Takegahara et al., 2022). However, recent research brought to light an unexpected role for this signaling pathway, revealing the existence of RANKL reverse signaling. This was initially observed through the use of RANKL-binding peptides which inhibit RANKL-induced osteoclastogenesis, but additionally resulted in increased bone formation *in vivo* (Takasaki et al., 1997; Aoki et al., 2006; Furuya et al., 2013; Sawa et al., 2018; Cheng et al., 2004; Kato et al., 2015), indicating the presence of a pathway where membrane-bound RANKL acts as a receptor to stimulate osteoblast differentiation. Deeper investigation into the putative ligand able to promote bone formation through RANKL identified this factor as the osteoclast transmembrane receptor RANK (Ikebuchi et al., 2018). Indeed, it was shown that maturing osteoclasts can secrete small EVs containing RANK, that activate the pathway (Ikebuchi et al., 2018). Vesicular RANK can bind to RANKL on the osteoblast surface and promote bone formation by activating the PI3K–Akt–mTORC1 pathway and triggering the production of RUNX2. The relevance of this mechanism was confirmed by the observation that suppression of RANKL reverse signaling leads to inefficient bone formation *in vivo* (Ikebuchi et al., 2018).

At the end of the resorptive phase, osteoclasts can undergo apoptosis and release apoptotic bodies whose function remain largely unknown. Curiously, a study revealed that apoptotic bodies from osteoclasts contained RANK and were able to stimulate RANKL reverse signaling, promoting mineralization by osteoblasts *in vitro* (Ma et al., 2021). This raises the question of why bisphosphonates, inducing osteoclast apoptosis, do not elicit a similar bone formation response when administered *in vivo*. Further studies are needed to elucidate this intriguing phenomenon within a physiological context.

## Clastokines in bone diseases: potential biomarkers and therapeutical targets

The discovery that osteoclasts release multiple factors to promote bone formation and preserve bone homeostasis provides not only a deeper understanding of bone biology but also holds the potential for clinical applications in the diagnosis and therapy of bone diseases.

Therapies for metabolic bone diseases, such as osteoporosis, aim to increase bone mass by utilizing anti-resorptive or anabolic treatments to promote bone formation. Ideally, an effective treatment would target both mechanisms, considering the coupling between bone formation and bone resorption. However, currently approved drugs tend to focus on one singular mechanism, often destabilizing normal bone homeostasis in the process.

With over 50 years of clinical use (Bassett et al., 1969; Russell, 2011), bisphosphonates are the current treatment strategy to block bone resorption by disrupting osteoclast function and survival (Rogers et al., 2011; Reyes et al., 2016). While they effectively control bone resorption, slightly increase bone density, and reduce fracture risk, bisphosphonates also strongly disrupt the bone repair mechanism (Nyman et al., 2004). This can give rise to clinical complications such as osteonecrosis of the jaw and atypical femoral fractures (Carvalho et al., 2011; Koh et al., 2010; Khan et al., 2009). Similarly, PTH, the only approved anabolic drug for the treatment of osteoporosis together with the anti-sclerostin antibody Romosozumab (Lindsay et al., 2016), promotes bone formation, but also leads to increased bone resorption as a secondary effect (Silva et al., 2011).

In this scenario, clastokines present an appealing therapeutical target as they can simultaneously stimulate bone formation and reduce bone resorption. Proof-of-concept evidence and promising results from *in vivo* administration of clastokines such as SLIT3 and vesicular RANK in murine models suggest their potential in alleviating bone loss in metabolic bone diseases. Also, multiple reports indicate the possibility to use them as biomarkers to predict the susceptibility to fracture or disease.

**Figure 3.**
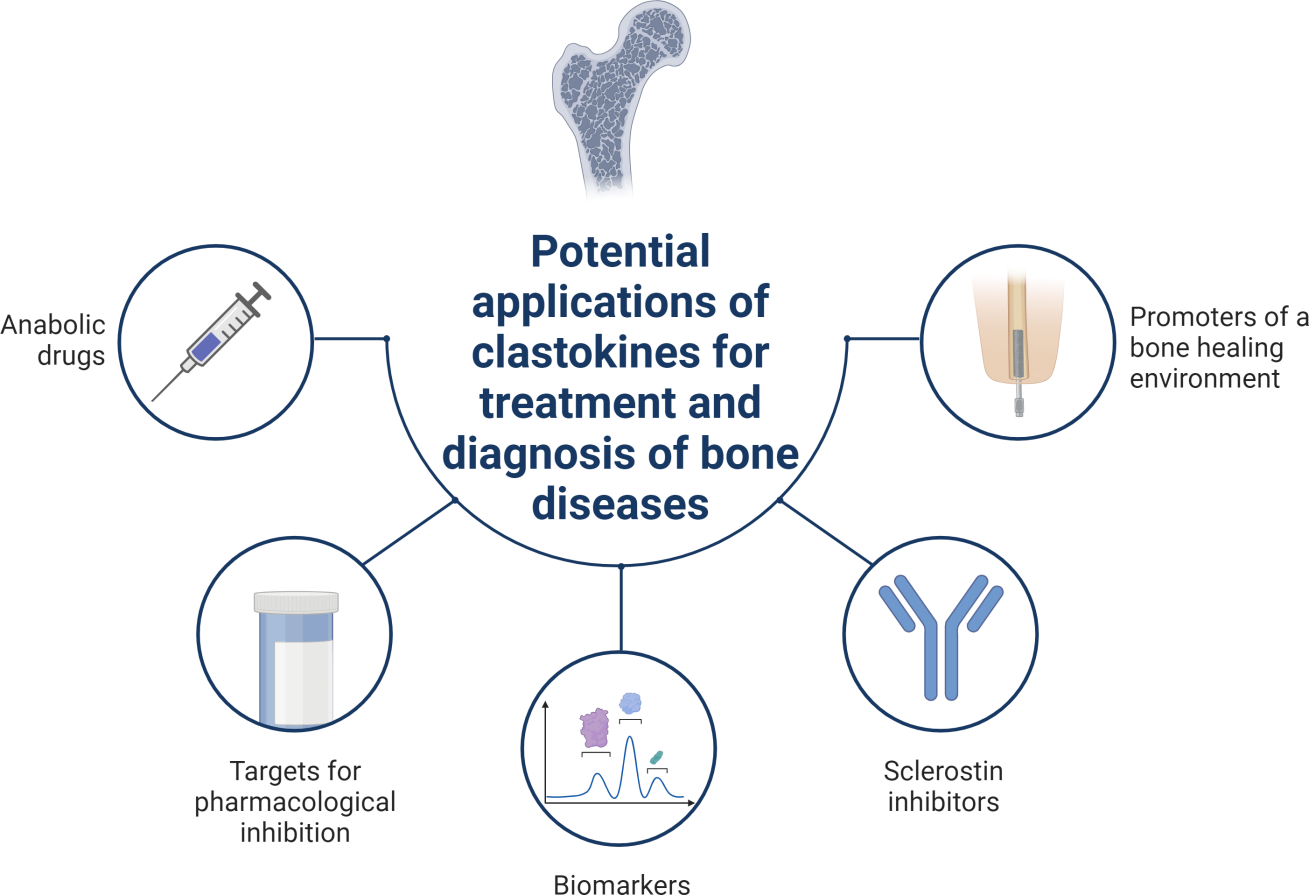
Schematic summary of potential applications of clastokines for treatment and diagnosis of bone diseases based on research evidence. Clastokines may be used as anabolic drugs able to promote bone formation without negatively affecting bone resorption. RANKL represents a target for pharmacological inhibition to activate reverse signaling and promote bone formation while simultaneously suppressing bone resorption. Circulating levels of clastokines correlate with bone quality in human, implying the possibility of using them as predictive biomarkers. Clastokines such as cardiotrophin-1 (CT-1) and leukemia inhibitory factor (LIF) have been shown to inhibit sclerostin production by osteocytes and have the potential to be used as sclerostin inhibitors with limited side effects. Titanium osteoimplants have been demonstrated to modulate osteoclast secretory phenotype, increasing clastokine production and promoting a bone healing environment.

Sclerostin, a natural inhibitor of the canonical WNT pathway and subsequently of bone formation, is a target for therapeutic intervention (Delgado-Calle et al., 2017). An anti-sclerostin antibody is currently FDA approved as a treatment for osteoporosis and rare genetic diseases such as osteogenesis imperfecta (Fabre et al., 2020; Aditya and Rattan, 2021; Marom et al., 2020), and the administration of PTH itself can reduce sclerostin levels (Keller and Kneissel, 2005). However, concerns have been raised about the possibility of serious adverse cardiovascular events following sclerostin inhibition (Pietrzyk et al., 2017; Bovijn et al., 2020; Kawaguchi, 2020), and anti-sclerostin antibodies are generally contraindicated in case of a history of myocardial infarction or stroke (Krupa et al., 2023). Interestingly, both CT-1 and LIF clastokines were reported to strongly inhibit expression of sclerostin by osteocytes (Walker et al., 2010). This could imply the possibility to use these coupling factors as therapies without the risk of adverse events associated with the use of anti-sclerostin antibodies (Figure 3). However, further studies are required to determine the applicability of this approach. Another intriguing finding is the increased secretion of LIF by osteoclasts during early abnormal bone remodeling in an unstable murine model of osteoarthritis. This observation opens the possibility of utilizing this factor as a biomarker or therapeutic target for the disease (Zhao et al., 2022; Figure 3).

Human recombinant SLIT3 LRRD2, a truncated form of SLIT3 composed of 130 amino acids, was shown to stimulate osteoblast migration and proliferation by inducing the release of *N*-cadherin-associated β-catenin, similar to full-length SLIT3. Furthermore, LRRD2 was found to associate with ROBO1 and ROBO2 receptors in osteoblasts, and ROBO1 and ROBO3 receptors in osteoclasts, thus exerting the same signaling events as its full-length form. Interestingly, ovariectomized mice intravenously injected with LRRD2 demonstrated significantly improved bone mass and bone parameters (Kim et al., 2018). These findings are particularly significant as SLIT3 represents a very appealing potential therapy for metabolic bone diseases due to its osteoprotective nature, inhibiting bone resorption while stimulating bone formation. Furthermore, significant progress has been made in the potential use of SLIT3 as a biomarker. Elevated plasma levels of SLIT3 were found to correlate with higher bone mineral density at the lumbar spine and proximal femoral sites in a large cohort of postmenopausal women. This suggests that circulating SLIT3 could serve as a valuable biomarker for predicting bone health in humans (Kim et al., 2018; Figure 3).

Similar to SLIT3, RANKL is an intriguing pharmacological target for simultaneously stimulating bone formation and inhibiting bone resorption. Researchers identified a bifunctional antibody (αR-bif) capable of inhibiting RANKL forward signaling while activating RANKL reverse signaling. The peptide, administered in a murine model of postmenopausal osteoporosis, partially rescued the phenotype by suppressing osteoclast differentiation and simultaneously stimulating bone formation (Furuya et al., 2013). The humanized monoclonal antibody Denosumab, blocking RANKL and exerting potent antiresorptive activity, is currently used as a treatment for osteoporosis (Cummings et al., 2009; McClung et al., 2006; Lewiecki et al., 2007). Contrarily to bisphosphonates, long-term treatment with Denosumab has shown to sustain a continued increase in bone mineral density, pointing out a unique mechanism of action of the drug (Törring, 2015). Recent hypotheses suggest that Denosumab may trigger RANKL reverse signaling thus stimulating an anabolic response in osteoblasts (Portal-Núñez et al., 2017). If validated, this would not only demonstrate the relevance of the signaling pathway in a human physiological context but also support the clinical applicability of targeting this coupling mechanism (Figure 3).

The observation that not all clastokines are necessarily expressed by mature osteoclasts indicates that osteoclasts can release coupling factors to promote bone formation while not actively resorbing bone. Studies conducted on osteopetrotic animals confirmed that induction of bone formation by osteoclasts can occur independently of their resorptive ability (Karsdal et al., 2007; Segovia-Silvestre et al., 2009; Del Fattore et al., 2006). This concept carries significant clinical implications since targeting osteoclastic bone resorption, rather than osteoclast differentiation, could be pursued without affecting bone formation. In this context, the use of cathepsin K inhibitors, in contrast to antiresorptive agents, represented a promising approach as they reduce osteoclast function while maintaining or even increasing the number of osteoclasts that retain their ability to secrete coupling factors and communicate with osteoblasts to promote their function and activity (Drake et al., 2017). Interestingly, cathepsin K inhibition has a stimulatory effect on S1P clastokine production (Lotinun et al., 2013). However, since cathepsin K function is not limited to bone, but is also present in brain, cardiovascular system, and lungs (Dai et al., 2020), its inhibition can have detrimental side effects on these tissues (Drake et al., 2017). This raised concerns limiting the development of new inhibitors and withdrawal of the cathepsin K inhibitor Odanacatib from a phase III clinical trial due to an increased risk of cerebrovascular accidents (Chen et al., 2023). Nevertheless, targeting bone resorption without compromising osteoclast number remains an alternative strategy to bisphosphonates that can be explored to take advantage of the coupling effect in the context of bone diseases.

Intriguingly, recent research showed titanium nanotubular surfaces with the ability to modulate macrophage and osteoclast secretory profiles toward the production of pro-healing cytokines and pro-osteogenic clastokines such as BMP6, CTHRC1, HGF, SLIT3, and WNT10B, respectively (He et al., 2022). This significant discovery paves the way for the potential application of titanium osteoimplants able to accelerate bone regeneration through clastokine production and represent a proof-of-concept for their future translation into the clinic (Figure 3).

Taken together, multiple pieces of evidence show clastokine potential for the treatment of bone diseases. The application of new refined technologies will likely help to decipher the specific mechanisms governing clastokine anabolic activity and understand how to translate such information into therapeutic solutions with clinical applicability.

## Conclusions and future perspectives

**Figure 4.**
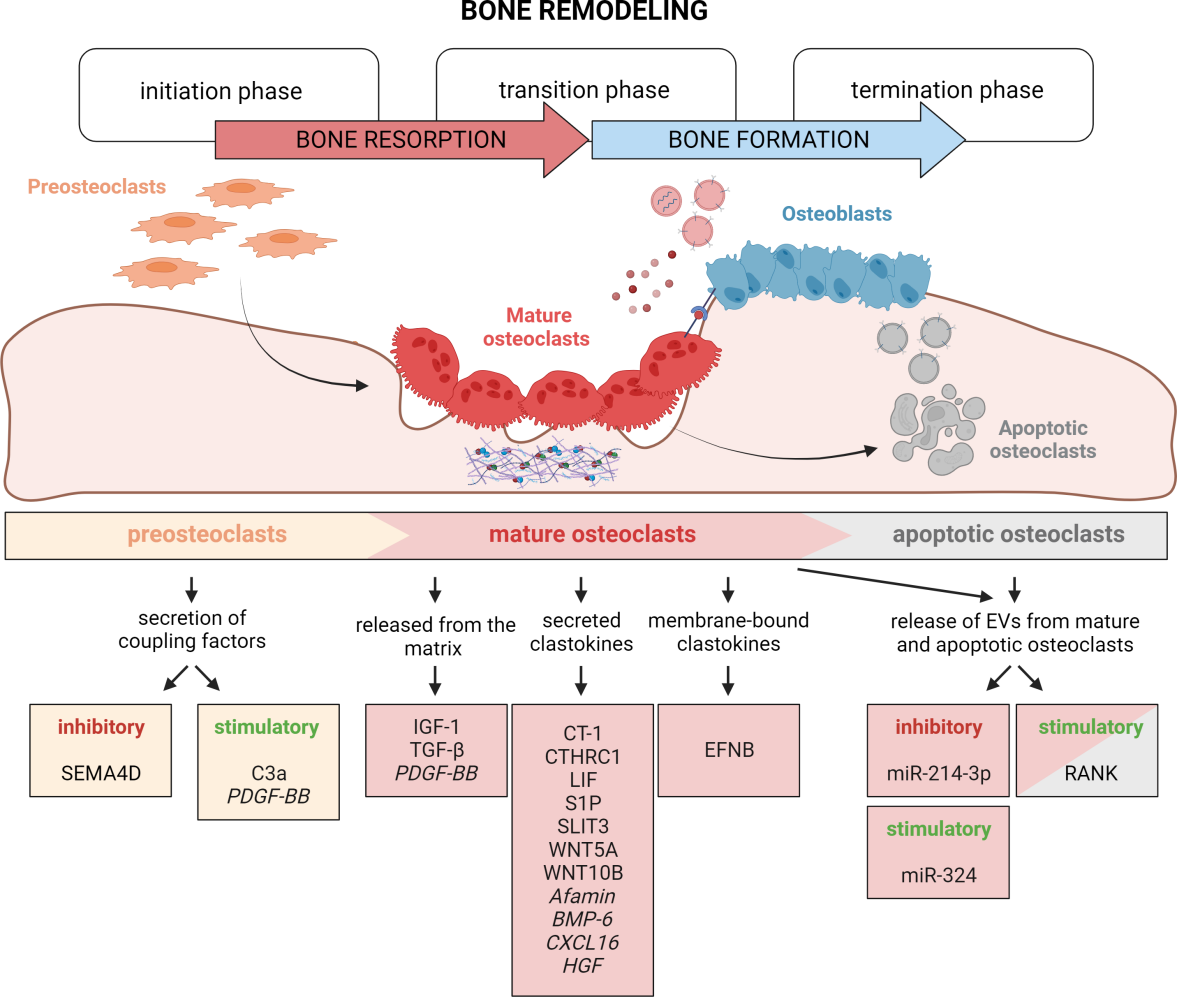
Timely activation of clastokines during bone remodeling. Bone remodeling is generally classified into three phases: initiation, transition, and termination. During the initiation phase, preosteoclasts are recruited to the bone remodeling site. These cells have been demonstrated to secrete C3a and PDGF-BB clastokines, but also soluble SEMA4D, which has an inhibitory effect on osteoblast differentiation. In the transition phase, mature, bone resorptive osteoclasts promote switching toward bone formation in three ways: by releasing coupling factors from the matrix, by directly secreting them, and by engaging in direct cell–cell contact with cells of the osteoblast lineage. They are also able to release extracellular vesicles (EVs) containing stimulatory and inhibitory miRNAs or vesicular RANK, activating RANKL reverse signaling. Apoptotic osteoclasts have been shown to also release vesicular RANK, which could further stimulate osteoblast differentiation during the termination phase. Putative clastokines are listed in italic.

Osteoclasts are not inert spectators of the BMU and work in concert with osteoblasts to preserve bone homeostasis. During bone remodeling, osteoclasts play a key role in the switch from bone resorption to bone formation (Figure 4). This can be achieved by their active release of factors from the extracellular matrix, engaging in direct cell–cell contact and, most intriguingly, by secretion of coupling factors known as clastokines that signal through osteoblast receptors.

The discovery of clastokines, although challenging, has sparked significant interest in the field. Continuous advancements in technology have facilitated the identification of new potential clastokines (Weivoda et al., 2020), while further research has revealed the molecular mechanisms of already known clastokines during human bone remodeling (Borggaard et al., 2022), emphasizing their conserved role in maintaining bone homeostasis and evidencing how large-scale genetic screening combined with bioinformatic prediction, and exploration of the osteoclast secretome are powerful approaches to efficiently identify such factors in humans.

Despite the efforts made in unraveling the intriguing world of clastokines, a lot of open questions remain about the mechanisms and pathways governing clastokine function and therefore there is a compelling need for more comprehensive investigation to provide a deeper understanding of clastokine function.

Osteoclasts, mostly in the mature stage but also independently of their differentiation, can positively stimulate osteoblast activity. The recent discovery of recycling osteomorphs, a new osteoclast type involved in the regulation of bone remodeling, raises an intriguing question: could there be a distinct osteoclast subtype with the specialized capacity to secrete clastokines and orchestrate osteoblast differentiation? And if so, how is it distinct from other types of osteoclasts? This interesting possibility requires further exploration and use of recently developed single-cell technologies could hold the key to answering this question.

The *in vitro* study of osteoclast-derived clastokines has a lot of space for technological improvement as the way that research has been carried out has not changed much with time. For instance, the use of conditioned media from osteoclasts to induce osteoblast differentiation could be replaced by direct co-cultures, which could not only address the presence of secreted factors, but also identify mechanisms of direct cell–cell communication, which are yet poorly explored and understood. Recent work using co-cultures of cells expressing Ephrin A1 and its receptor EPHA2, respectively, has nicely shown the trafficking of Ephrins through live cell imaging (Valenzuela and Perez, 2020). A similar approach could be applied to study clastokine secretion and trafficking between osteoclasts and osteoblasts.

There is also a strong need for *in vivo* validation of *in vitro* data. Many murine models used for the study of clastokine function are global knockouts, demanding the need to study osteoclast-specific knockouts to show the contribution of osteoclast-secreted factors to the observed phenotype. Challenges arise also from the fact that the most widely used osteoclast driver, Ctsk-Cre, has recently been identified to be active in various other skeletal cell types including osteoblasts, making it difficult to specifically associate the observed phenotype to the osteoclast per se and complicating the interpretation of the results (Debnath et al., 2018; Ruiz et al., 2016; Chai et al., 2023). In this light, the use of LysM-Cre, targeting the myeloid lineage, offers an alternative strategy to conditionally delete gene function in myeloid cells including osteoclasts. The combinatorial use of Cre and Dre recombinases would also represent a more refined way to achieve cell specific and efficient genetic targeting for the study of osteoclast function (Wang et al., 2022), fundamental in the field of clastokine research. The non-tissue-specific expression of some of the transgenes can be traced back to the specific promoter fragment used or local effects at the integration site of the transgene (Michalski and Williams, 2023). New genome editing techniques based on the Clustered Regularly Interspaced Palindromic Repeat CRISPR/Cas system open up the possibilities to efficiently modify the endogenous gene locus, circumventing some of the previously mentioned limitations of other transgenesis strategies (Gurumurthy et al., 2019; Ohtsuka and Sato, 2019).

While we have a better-defined picture of how clastokines work in a physiological context, our knowledge regarding their role in bone diseases remains limited. It is widely accepted that metabolic bone diseases are associated with a disruption in osteoblast to osteoclast communication. However, if this is also true for osteoclast to osteoblast communication remains an open question. Knowledge about how clastokine secretion by osteoclasts is affected in a pathological context, would allow the use of clastokines as biomarkers of disease states and provide opportunities to target their function to develop specific therapies.

Unquestionably, a growing body of evidence supports the therapeutic potential of clastokines for the treatment of bone diseases. The tight coupling between bone resorption and bone formation still represents nowadays an obstacle to the promotion of bone anabolism by singularly targeting either mechanism. Clastokines, owing to their osteoprotective role and their ability to couple bone formation and resorption toward bone anabolism, hold promise as therapeutic agents to overcome this limitation. The osteoprotective functionalities of S1P and SLIT3, the latter already demonstrated to act as an anabolic drug *in vivo*, represent novelties with great clinical potential. The discovery of RANKL reverse signaling mediated by osteoclast-derived vesicular RANK represents an interesting pharmaceutical target to bidirectionally stimulate osteoblasts and inhibit osteoclast differentiation and will be worth further attention. Notably, the paracrine signaling of osteoclasts is independent of their resorptive state, allowing for the selective targeting of bone resorption without disrupting osteoclast differentiation and consequently the coupling mechanism, thus offering another possible strategy for targeted therapies.

In conclusion, this review highlights the pivotal role of osteoclasts in orchestrating the dynamic interplay between bone resorption and formation. Significant steps forward have been made in identifying new clastokines and their anabolic effect over the past decades. The upcoming years are expected to bring further advancements, providing deeper insights into the mechanisms that these molecules adopt to preserve bone homeostasis. It is anticipated that, with the application of innovative technologies and strategies, the expanding field of clastokines and coupling factors will eventually translate into new clinical applications for the treatment of metabolic bone diseases.
